# Development of strategies to improve care for all in the communities served by a mid-size nonprofit health system in the United States

**DOI:** 10.3389/fpubh.2025.1614507

**Published:** 2025-08-01

**Authors:** Jodi R. Cichetti, Stephanie Voight, Jenna Jansen, Michael B. Seim

**Affiliations:** WellSpan Health, York, PA, United States

**Keywords:** quality improvement, community health services, life expectancy, health disparities, community health improvement plan, social drivers of health

## Abstract

WellSpan Health is a nonprofit integrated healthcare system serving 12 counties in south-central Pennsylvania and northern Maryland, a region marked by wide disparities in the demographic, social, and economic conditions of its people. Consequently, it is also marked by wide disparities in health status and health outcomes, including life expectancy. Beginning in the mid-1990s, WellSpan’s community health needs assessments identified numerous potential drivers of health disparities, and in recent years the hospital system has conducted increasingly sophisticated community health improvement plans to address disparities in ways compatible with the hospital system’s resources, capabilities, mission, and priorities. Because there is limited published information about how nonprofit health systems are implementing and evaluating community health improvement plans, we have documented key aspects of WellSpan’s progress including guiding principles and strategies, how pilot projects were identified and conducted, how community partnerships were developed and leveraged, and how data sources were used to guide decisions. WellSpan’s efforts have culminated in the recent adoption of a 30-year plan to improve overall life expectancy and quality of life and reduce disparities in these outcomes in the region served. The purpose of this paper is to share experiences and lessons learned during the multi-year effort leading to the development of this 30-year plan.

## 1 Introduction

People from marginalized and vulnerable groups in the United States have substantially worse health outcomes and lower life expectancy than people from other groups (1–4). These disparities are strongly influenced by social drivers of health such as the physical environment, access to clinical care, health behaviors, discrimination, and economic factors (1, 3–5). Local hospitals and health systems play a vital role in reducing health disparities by identifying the causes of poor health outcomes in the communities they serve, and by implementing strategies to address those causes. The Patient Protection and Affordable Care Act and changes to Internal Revenue Service Code 501(r) (3) require nonprofit hospitals to conduct a community health needs assessment (CHNA) every 3 years, and to develop and implement a community health improvement plan (CHIP) designed to address the needs identified by the CHNA (6). Those activities provide an opportunity for nonprofit hospitals to align their CHIPs with goals related to exceptional care for all and life expectancy, consistent with long-established missions of many nonprofit hospitals to improve the health of the individuals and communities they serve (7).

Recently published overviews found that nonprofit hospitals were highly compliant with requirements to conduct CHNAs, but many CHNAs lacked an evaluation of the effectiveness of programs implemented to address needs and health inequities (8, 9). These findings are consistent with earlier observations noting the limited information available about how hospitals are implementing and evaluating strategies to improve community health (10). The lack of published information about how nonprofit hospitals are implementing and evaluating community health strategies indicates that there is an ongoing need for publication of real-world examples of these activities, how hospitals put guiding principles into practice, how community health priorities are selected, how resources are assessed and deployed, and how community partnerships are formed and leveraged.

WellSpan Health, a mid-size nonprofit health system serving 12 counties in south-central Pennsylvania, has recently adopted a 30-year plan to address health disparities in the communities we serve. The plan evolved from WellSpan’s extensive experience conducting CHNAs and more recent experience developing, implementing, and evaluating CHIPs to address identified health disparities. In the current paper, we begin by describing the guiding principles and operating structure of WellSpan’s still-evolving “Exceptional Care for All” program. We then chronicle how those principles and structures were implemented during the development and evaluation of pilot projects addressing specific health disparities in our communities. Finally, we discuss how the lessons learned from those pilot projects culminated in the 30-year plan focused on improving healthcare for everyone.

## 2 Operational model and guiding principles for exceptional care for all improvement plans

WellSpan’s efforts to develop CHIPs focused on ‘health for all’ principles were initially guided by Achieving Health Equity: A Guide for Health Care Organizations from the Institute for Healthcare Improvement (IHI) (11). This guide describes key activities for achieving a stable, ongoing plan to improve care for all: (1) Make health equity a strategic priority, (2) Develop structure and processes to support health equity work throughout the organization, (3) Deploy specific strategies to address the multiple determinants (drivers) of health on which health care organizations can have a direct impact, and (4) Develop partnerships with community organizations to improve health, access to care, and health equity.

### 2.1 IHI key activities 1 and 2: institutional support, structure, processes, and governance

**Figure 1:**
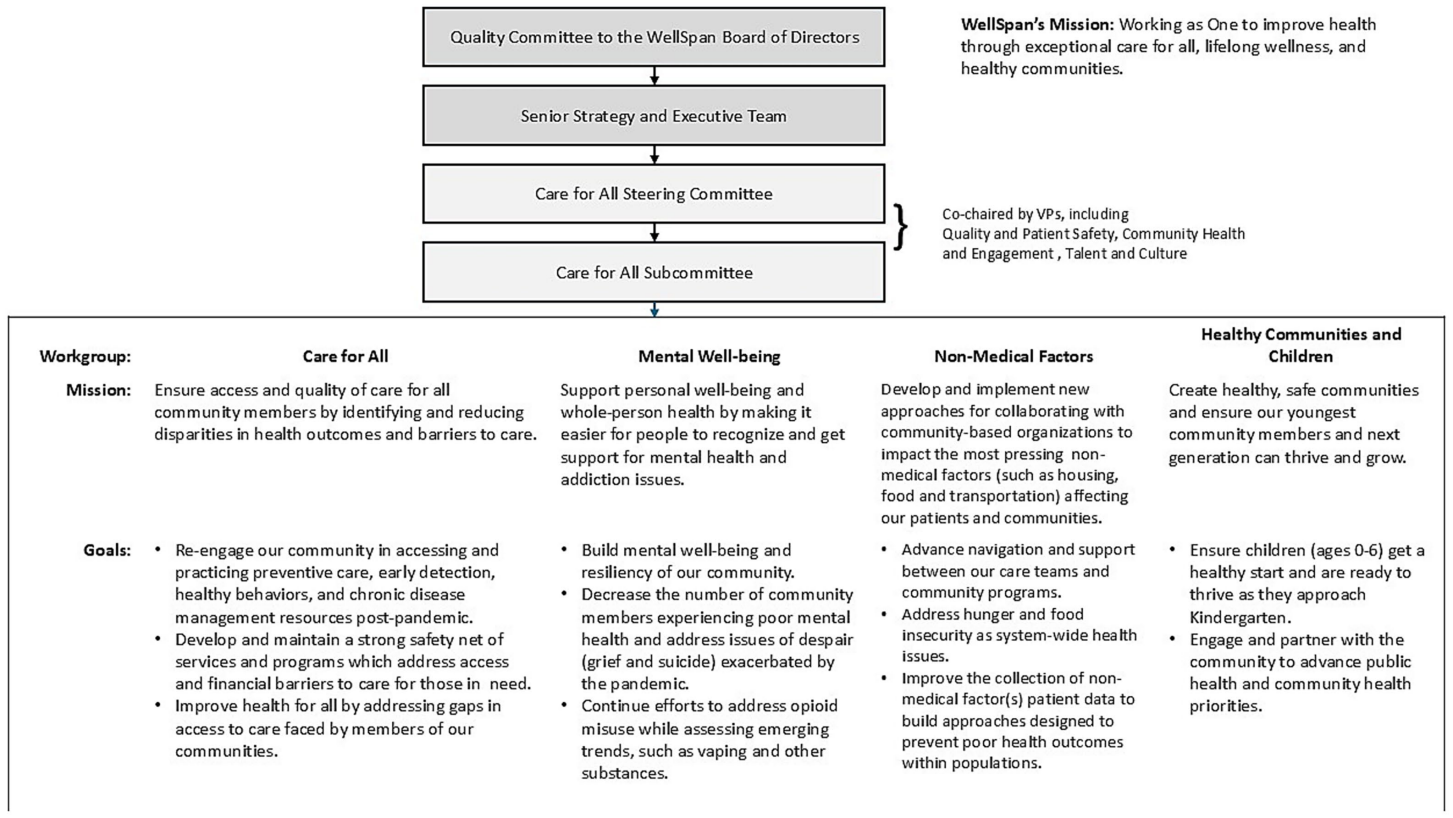
WellSpan’s “Care for All” governance structure, including workgroup missions and goals.

As noted by IHI Key Activity 1, an effective program requires full and ongoing support from the hospital’s governing and executive teams, as well as alignment with the health system’s mission and priorities. In practice, it is essential that the teams responsible for developing and enacting a CHIP have a clear position in the hospital’s governing structure, with defined bi-directional channels of communication between various teams. The governance structure adopted by WellSpan Health for development and implementation of a community strategy to provide exceptional care for all is shown in Figure 1, along with the mission of each of 4 designated workgroups.

**Figure 2:**
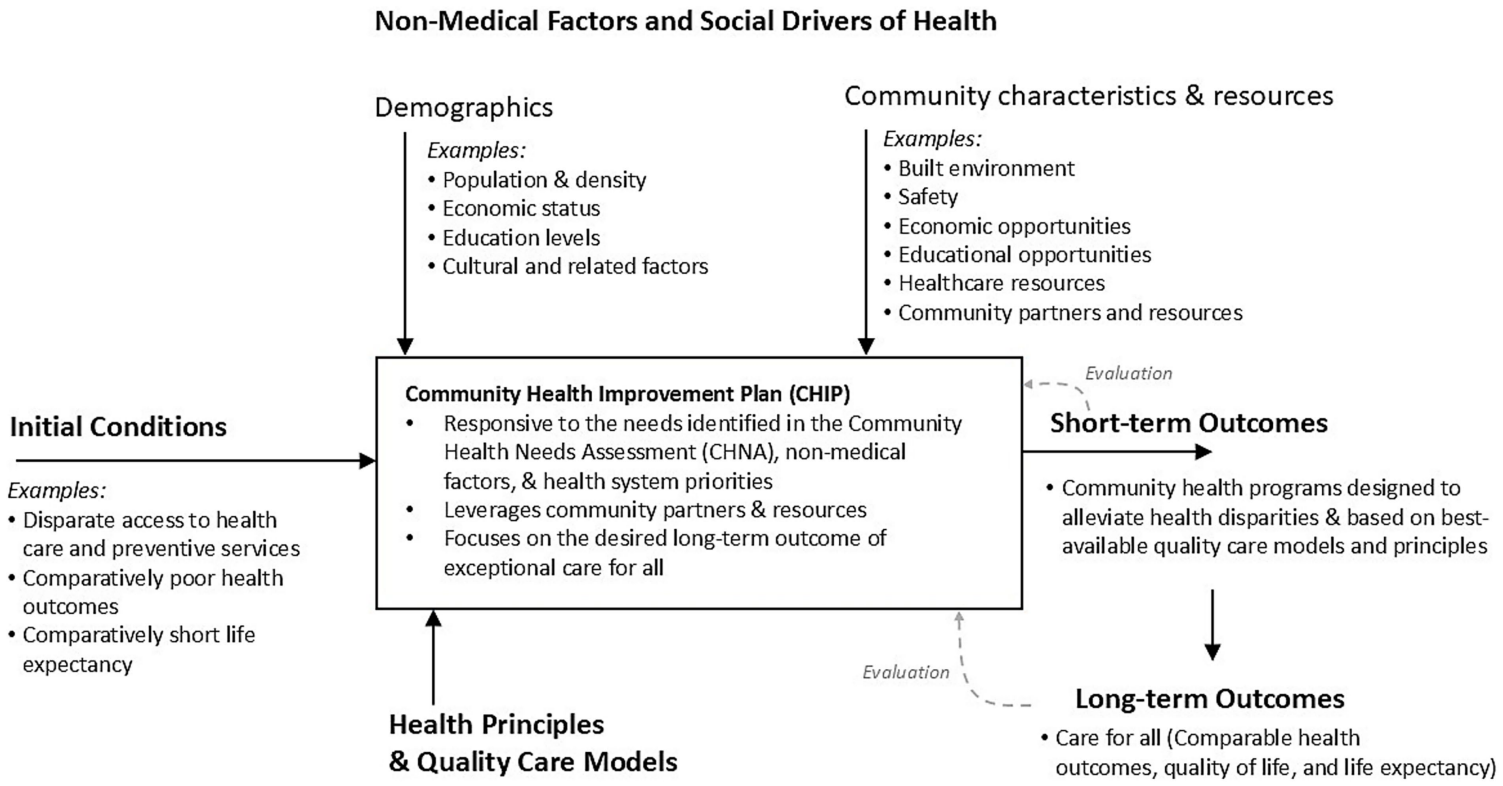
Schematic diagram of WellSpan’s process for developing a Community Health Improvement Plan, depicting how CHIP development activities were guided by initial conditions in the community, social drivers of health, guiding principles, and desired outcomes.

IHI Key Activity 2 involves developing structures and processes to support work that improves the health of people who have historically struggled to obtain healthcare. Figure 2 depicts WellSpan’s process for developing CHIPs focused on exceptional care for all. As shown, we aimed to address local initial conditions and social drivers of health to develop community health programs designed to ensure exceptional care for all and address poor health outcomes and short life expectancy in the communities we serve. Thus, WellSpan’s CHIP incorporates various quality-care models, leverages community partners and resources, and is aligned with WellSpan’s strategic operating priorities.

### 2.2 IHI key activities 3 and 4

Once the CHNA has identified drivers of health disparities, IHI Key Activity 3 recommends deploying specific strategies to address those drivers. Such strategies are identified during WellSpan’s planning process, and specific examples will be described below in Section 4.

#### 2.2.1 Community partners

Like all health care organizations, WellSpan has limited resources and capabilities, and thus is limited in its ability to implement community health interventions. WellSpan employs community health workers, social workers, and community-focused Registered Nurses (RN) who support the CHNA project, though our most effective means of engaging the community is by leveraging our relationships with community partners. Therefore, a key aspect of WellSpan’s CHNAs and CHIPs is collaborating with community partners, involved parties, and subject matter experts, as recommended in IHI Key Activity 4. Organizations and individuals actively collaborating with WellSpan in these activities included: behavioral health care providers, colleges & universities, county and municipal governments, faith-based organizations, federally qualified health centers, Area Agencies on Aging, community health coalitions, health care providers, philanthropic organizations, recreation centers (YMCA/YWCA), schools (elementary through high school), United Way, social/human service agencies, economic development organizations and businesses, community members, and volunteers. A complete list of community partners is beyond the scope of this paper, but key partners are 4 local health coalitions and 3 federally qualified health centers (FQHCs), which serve as umbrella organizations that convene more than 100 community partners in these efforts. Those 7 key partners are: Healthy Adams County, Healthy Franklin County, Community Health Council of Lebanon County, Healthy York Coalition, Union Community Care, Family First Health, and Keystone Health. Such partners not only augment WellSpan’s capabilities, but they are often essential for aligning the CHIPs with the values and wishes of the community, for communicating with community members, and for developing and maintaining the community’s trust.

#### 2.2.2 Relationships with community partners

When working with community partners, it is important for the CHIP to define the health system’s role so there is a clear action plan, duplicative efforts are avoided, and all involved organizations are aligned regarding expectations. To help define its role in various CHIPs, WellSpan followed guiding principles of The Advisory Board (www.advisoryboard.com), a for-profit public company providing consulting services to the healthcare industry. In its freely available Field Guide for Defining Roles in Addressing Social Determinants of Health (12), the Advisory Board recognizes five roles for a health system seeking to drive change:

1. Funder – Devote staff and resources; provide grant support
2. Convener – Recruit parties for collaboration; Build channels for communication
3. Expert – Contribute existing knowledge; Conduct studies to build academic evidence base
4. Advocate – Engage policymakers; Publicize system policy positions
5. Anchor – Contract with local businesses; Invest in the workforce

WellSpan’s role varies by community, by identified care or driver of need, by available resources and partners, and by the type of intervention. An example, described in more detail in Section 4, is WellSpan’s breast cancer screening program. WellSpan has partnered with churches and other community organizations to provide this service to community members who may otherwise have limited access. In this instance, WellSpan dispatches a mobile breast cancer screening unit, acting as expert and funder by providing staff and resources to operate the service. WellSpan also acts as a convener by engaging and recruiting community partners and helping them communicate the importance of breast cancer screening to community members. The partners advertise the event and engage and communicate with community members, thereby increasing community knowledge about the importance of screening. Partners also help establish and maintain trust between community members and WellSpan.

#### 2.2.3 Understanding with community partners

WellSpan’s CHNA process actively engages the local health coalitions and FQHCs mentioned in Section 2.2.1. The executive directors of the four local health coalitions are employed by WellSpan, and WellSpan is their fiduciary backbone. The 4 executive directors, along with the system director of WellSpan Community Health, make up the Steering Committee for the CHNA. At least quarterly (every 3 months) throughout the project timeline, the coalition leaders engage their steering/advisory committees and aligned task forces to solicit feedback related to WellSpan’s CHNA-CHIP cycle, including methods, planning, preliminary findings, priorities, and dissemination of findings. The WellSpan CHNA project timeline incorporates these periodic pauses to ensure feedback is received and incorporated. Once CHNA and CHIP priorities are identified, the coalitions utilize local and regional reports to develop strategic plans as well as annual Memoranda of Understanding (MOU) between WellSpan and the coalitions. The MOUs center around key deliverables identified in the WellSpan CHIP plan. In some cases, the coalitions collaborate with other health systems within their county to ensure synergy and consistency in the identified priorities.

## 3 The community health needs assessment

The purpose of a Community Health Needs Assessment (CHNA) is to understand the most pressing health-related challenges in the community. A CHNA should also identify resources that promote health and wellness, and gaps in those resources. It should also ensure community organizations, including healthcare providers, are equipped with the data needed to take measurable action to address community health needs. One rationale for the requirement to conduct a CHNA is to ensure not-for-profit hospitals are providing community benefits that justify their tax-exempt status (10, 13). CHNAs (and CHIPs) are also important tools for advancing the mission and values of not-for-profit hospitals. The requirements also reflect a policy goal of the Affordable Care Act: to shift health care in the United States toward a prevention-based system and away from a reactive acute-care system (10).

In 2011, a forum sponsored by the Centers for Disease Control and Prevention (CDC) explored current and potential practices for conducting CHNAs and for development of implementation strategies (14). The CDC followed up by providing technical assistance and resources for hospitals to conduct CHNAs and develop implementation strategies (15), including guidance from external experts regarding key principles and practical steps of this process (see for example references (16, 17)). Because of such readily available technical information and support, the current paper will not address technical aspects of conducting a CHNA. However, specific topics related to WellSpan’s CHNA process will be discussed in more detail because they are relevant to the current paper.

### 3.1 CHNA data sources and analysis

WellSpan’s CHNA uses data from numerous sources including patient data; provider surveys; data extracted from local, state, and national sources; a representative community survey available in both English and Spanish; a special populations survey intended to capture more data from traditionally under-represented groups; and other efforts to capture data from special populations—efforts such as focus groups and informant interviews. During the period described in this paper, WellSpan had a significant market share in 5 counties in south-eastern Pennsylvania (Adams, Franklin, part of Lancaster, Lebanon, and York), so our discussion is relevant to CHNAs that covered those counties.

WellSpan’s CHNA collects primary data at the household level. Our CHNA survey responses are representative of the demographic attributes of our community as defined by the US Census. This has been achieved by collaborating with Franklin and Marshall College’s Center for Opinion Research, which utilizes a multimodal strategy to reach demographic targets for the survey. Calibration-focused statistical methods are utilized to ensure that the demographic characteristics of the respondents are representative of the community.

WellSpan utilizes community relationships, social media and other methods to expand the reach of our primary survey and increase the sample size. This mixed-methods approach does not permit the identification of a true response rate for the sample, but the total number of responses far exceeds the minimum required sample to achieve statistical significance.

It is expected that organizations conducting CHNAs will implement ongoing improvements in the CHNA process, due to evolving research methods and data sources. A key goal of such efforts is to improve how well the CHNA represents the community, including how well it captures the needs of historically underrepresented groups. WellSpan has been conducting needs assessments for decades and continues to improve its processes, including the addition of off-cycle assessments to gain additional insight into special populations, such as the Plain community (18). Additional strategies, such as incorporation of patient data from electronic health records and oversampling of specific geographic regions, have meaningfully contributed to WellSpan’s CHNAs for many years.

Since 2018, WellSpan has used electronic health records to collect information about non-medical factors and social determinants of health (SDOH), including food and housing insecurity and access to transportation. These data are collected for children and adults in inpatient and ambulatory settings using both staff-patient interactions and WellSpan’s online patient portal. Beginning in 2023, WellSpan implemented a closed-loop referral platform to seamlessly refer patients with social insecurities to the most appropriate internal resources and/or external agencies, and to monitor the impact of such referrals. There have been more than 1 million patients screened for social insecurities (as of report Jan 1, 2025). In 1 year of implementation of the closed loop referral system, WellSpan has facilitated more than 11,000 referrals to appropriate community resources. In addition, part of our CHNA-CHIP cycle involves annual reviews of progress in achieving CHIP goals through both our Community Benefit Report and an internal CHIP reflection report.

## 4 Putting it into action: examples of WellSpan’s experience developing and implementing community health improvement plans

Since the mid-1990s, WellSpan’s CHNAs revealed numerous potential drivers of poor health outcomes (relative to regional or national outcomes) in the communities served. Recent CHNAs have revealed high numbers of uninsured individuals, low rates of colon cancer screening, low rates of breast cancer screening, high rates of mental health problems, food and housing insecurity, financial stress, low rates of physical activity, and other drivers. As a starting point, in 2018 WellSpan began focusing on individual disease states in which we could meaningfully improve the comparatively poor health outcomes in the community. Since then, WellSpan’s CHIPs have evolved into more strategic plans to address health disparities, and eventually to a broader, multicomponent, 30-year plan to improve overall life expectancy and reduce disparities in life expectancy in our community. This evolution is outlined in Table 1, and examples of selected projects are described in detail in the following subsections. Note that most of this work was conducted when WellSpan served only five counties, so most of the analyses include only those five counties.

**Table 1:** Evolution of WellSpan’s strategy and specific community health improvement projects.

Year initiated	Strategy/Goal	Projects
2018	Focus on a single disease state in which WellSpan can have impact.	Treatment of maternal hypertension
	Understand social drivers of health in the community.	Collect and analyze data on social drivers of health. What are the social drivers of health in our community? What are the demographic characteristics of the community? Mapping: Where do people live who are adversely affected by social drivers of health?
2021	Expand focus to address high rates of selected preventable illnesses.	Increase Breast cancer screening rates Increase Colon cancer screening rates
2022	Expand list of illnesses for intervention planning.	Hypertension management
	Initiate a 30-year strategic goal to eliminate disparities in life expectancy in our communities.	Death analysis in each WellSpan community
2023	Continue to expand screening programs and intervention planning for illnesses with demonstrated disparities across various populations.	Increase kidney health screening in groups with historically low screening rates.
2024-Current	Decrease the disparity in cancer and hypertension screening rates across various populations.	Increase screening in groups with historically low screening rates and decrease the disparity in screening rates across groups.
	Enhance data collection and analysis capabilities to guide development and assessment of efforts aimed at improving life expectancy for various demographic groups.	Identify evidence-based drivers correlated with life expectancy and appropriate for a WellSpan CHIP. Develop two evaluation cohorts; one representing people who use the health system, another representing community members who may or may not use the health system. Establish a dashboard allowing easy visualization of key indicators in the two evaluation cohorts. Display and monitor demographic disparities in the context of how they affect life expectancy

### 4.1 Treatment of maternal hypertension

In 2018, we used Premier Quality Advisor (19) to analyze hospital discharge records of four WellSpan hospitals to explore how race, ethnicity, or payor (as a surrogate measure of financial status) were related to a woman’s risk of severe maternal morbidity (SMM). The definition of SMM was that used by the CDC as well as the Alliance for Innovation on Maternal Health (20, 21). Previous analyses of national data had shown major disparities in maternal morbidity and mortality across racial and ethnic groups, with especially high rates among Black women as compared with White women (22).

The results of our analysis of WellSpan’s records are shown in Supplementary Table 1 (Year ending July, 2019 columns). Women who identified as Black or African American had significantly higher rates of SMM than White women (*p* = 0.00435 by logistic regression), consistent with national trends (22). The most common diagnoses related to SMM at the WellSpan hospitals were pre-eclampsia and postpartum hypertension.

During subsequent strategic planning and CHIP development, this disparity was identified as a strategic priority for several reasons: 4 in 5 maternal deaths are preventable (23), WellSpan had the resources and capabilities to meaningfully impact treatment of maternal hypertension in outpatient clinics and immediately impact rates of hypertension-related complications, and best-practices for treatment of maternal hypertension were readily available (24, 25). Thus, WellSpan’s 2018 CHIP called for several interventions to improve management of maternal hypertension at WellSpan sites. Interventions included early recognition of maternal hypertension through screening, implementation of a best-practice protocol of low-dose aspirin therapy (starting as early as 12 weeks of gestation), and system-wide changes to the coding and documentation of maternal hypertension and pre-eclampsia.

Analysis of data for subsequent years (pooled data 1 July 2019 to 30 June 2022) found an overall 17% reduction in rates of SMM relative to baseline, including a 61% reduction in hypertension-related SMM among Black women (Supplementary Table 1, July 2019–June 2022 Columns).

Several lessons were learned during this project. First, the interventions were deployed only at WellSpan-operated outpatient sites; a more comprehensive community-based initiative may be required to reduce SMM rates in the broader community. Second, because the interventions focused specifically on treatment of maternal hypertension, the analysis may have been more sensitive to intervention effects had it focused only on hypertension-related SMMs. Finally, it may have been valuable to include an evaluation of intermediate outcomes —such as rates of compliance to guidelines for low-dose aspirin therapy when indicated—in the project plan. Such an intermediate evaluation may have allowed ongoing adaptation of the interventions to optimize their reach and effectiveness. The lessons learned from this effort will help inform the design of subsequent CHIPs.

### 4.2 Breast cancer screening

In WellSpan’s 2022 CHNA, several opportunities were identified to reduce rates of preventable illness in the communities we serve. For example, rates of recommended breast cancer screening and colon cancer screening were low, and management of hypertension was suboptimal among community members. For the limited scope of this paper, we will describe an initiative developed to improve rates of breast cancer screening in the community.

Analysis of patient records in August 2021 found that 70.26% of eligible patients (defined as women aged 50–74 years) had undergone breast cancer screening mammography within the preceding 27 months. As shown in Supplementary Table 2 (Baseline rows), screening rates were modestly lower among patients who self-identified as a race or ethnicity other than White, non-Hispanic. These findings were consistent with previous analyses of breast cancer screening rates among WellSpan patients, and they prompted efforts to understand barriers to breast cancer screening from the perspective of community members. Therefore, WellSpan and community partners collaborated to conduct outreach and listening sessions at several community venues such as churches and community centers. One of the topics discussed was barriers to breast cancer screening among eligible women. We heard from community members that many women were more likely to participate in screening if it were conducted at their church or community center because of the convenience and trust associated with their community center, and the cost and time commitment involved in going to a clinic or hospital for screening mammography. In addition to these needs, an analysis of patient records revealed that women who did not speak English had substantially lower rates of breast cancer screening than English-speaking women; this disparity was especially notable among Spanish-speaking women.

In response to these identified needs, we developed a multi-pronged approach with the goal of increasing breast cancer screening rates among WellSpan patients by addressing some of the identified barriers. Many of the same community partners were eager to help communicate the importance of breast cancer screening to their constituents and served as hosts for WellSpan’s mobile screening mammography unit. To address the disparity among Spanish-speaking women, WellSpan launched an outbound phone call program to contact Spanish-speaking women with the goal of increasing the number of Spanish-speaking patients completing screening mammography. Calls to Spanish-speaking women who were overdue for breast cancer screening were designed to educate them about the importance of regular screening, address any barriers to screening, and schedule an appointment. Between March, 2022 and September, 2024, the program identified 918 Spanish-speaking women overdue for their tests. Of these, 394 (43%) were successfully contacted via a phone call and agreed to schedule a mammogram, 197 completed mammography, and 15 (7.6%) had an abnormal finding. The Spanish language program recorded 125 h of employee time ($3,600). Early detection of these abnormal results prevented more invasive testing and/or treatment that would have cost an estimated $121,264, based on an analysis of insurance claims in the year after diagnosis and assuming 2 of those 15 patients ultimately had a diagnosis of stage 0–1 breast cancer (26).

In June 2024, the analysis of patient records was repeated to again determine the percent of eligible patients who had undergone screening mammography within the preceding 27 months (Supplementary Table 2, June 2024 rows). As shown in the table, the percentage of eligible women who were up-to-date with screening mammography increased during the project timeline. The percentage of women who self-identified into one of the non-White racial or ethnic groups also increased, but slightly less than the overall increase despite the apparent success of the Spanish language initiative. This finding suggests a need to redouble our efforts to identify and alleviate barriers to screening in these groups if we seek to eliminate disparities in health care access and outcomes.

## 5 Toward a 30-year plan to eliminate disparities in life expectancy

As this paper has documented, WellSpan has progressively expanded its commitment to understanding and addressing community health disparities, and its CHNAs and CHIPS have reflected that growing commitment. As outlined in Table 1, WellSpan’s CHIPs have evolved from isolated departmental initiatives to system-wide plans focused on specific preventable illnesses, and eventually to strategic plans aimed at improving life expectancy—and eliminating disparities in life expectancy—in our communities. This section will describe the background and rationale for that evolution.

### 5.1 Disparities in life expectancy across the region served by WellSpan

Five of the counties WellSpan serves in south-central Pennsylvania (Adams, Franklin, Lancaster, Lebanon, York counties) comprise diverse rural, suburban, and urban communities. An analysis of life expectancy across those counties revealed wide disparities across regions and across racial and ethnic groups (Supplementary Data Sheet 2). Furthermore, the average life expectancy in urban settings was markedly lower than in suburban settings. In York County, for example, one urban census tract had an average life expectancy of 67.8 years but a suburban tract only 6.7 miles away had an average life expectancy of 89.8 years.

### 5.2 Drivers of disparities in life expectancy

To begin understanding the drivers of these disparities, WellSpan analyzed 3–5 years of data from publicly available data sources including United States census records, public records of causes of death, health data from local health departments, WellSpan’s inpatient medical records, and reported social drivers of health. The analysis focused on establishing an understanding of the distribution of deaths by age, race and geography (defined by zip codes or municipalities), as well as causes of death. The analysis demonstrated a correlation between premature death and race or ethnicity: Black/African-American residents and other non-White residents were two- to three-times more likely to die before the age of 55 years than White residents. Leading causes of death for three age groups within WellSpan’s service area are shown in Table 2, and they are consistent with national trends. In geographic regions with higher rates of death among people under 55 years of age, there were also high poverty rates, low rates of home ownership, low educational attainment, and low median household income. These observations are consistent with WellSpan’s internal data, which showed strong correlations between social drivers of health and health outcomes.

**Table 2:** Leading causes of death in WellSpan’s service area according to age category.

Age 0–24 years	Age 25–54 years	Age 55 and above
Motor vehicle accidents (12.1%) Intentional self-harm (9.4%) Cardiovascular disease (3.9%) Homicide (3.4%)	Cardiovascular disease (17.3%) Diseases of the heart (14%) Intentional self-harm (7.7%) Motor vehicle accidents (5.0%)	Cardiovascular disease (31.5%) Disease of the heart (24.3%) Stroke (5.4%) Lung cancer (5.1%)

This analysis, along with findings from CHNAs, revealed evidence that disparities in life expectancy were associated with disparities in chronic disease prevalence, access to health care, and other social drivers of health. These findings are consistent with—though perhaps more severe than—findings reported elsewhere regarding health outcomes and health disparities in the United States (27–30). These reports have documented causes of disparities in life expectancy in the United States, not limited to poverty, lack of educational opportunity, limited access to food, housing, and transportation, high rates of chronic disease, and other social factors.

### 5.3 Identifying WellSpan’s role in efforts to reduce disparities in life expectancy

As described earlier with respect to Figure 1, health systems and their partners have limited resources and limited capacity to influence social drivers of health. For example, WellSpan and its partners have limited ability to modify poverty levels, educational opportunities, or other factors occurring outside of WellSpan’s programs and services. Nevertheless, some of the social drivers described in the preceding section are within WellSpan’s purview and addressing them is compatible with WellSpan’s capabilities and priorities. One example is the group of drivers falling under the umbrella of access to care. Indeed, WellSpan has consistently committed approximately 9% of its operating income to community benefit activities aimed at improving access to care, including grants, financial support of health coalitions within the service area, community programs addressing social drivers of health, subsidized health services, and mobile health. Since 2019, WellSpan’s community grants program has funded multiple community and health system initiatives to improve SDOH that affect quality of life and life expectancy. Within the last 5 years, WellSpan has supported grants totaling more than $10 million focused on food, housing, and transportation.

To identify potential programs for inclusion in a long-term plan to improve life expectancy—and reduce disparities in life expectancy—we performed an initial analysis of factors correlated with life expectancy in WellSpan’s overall service area as well as in localized regions. For this analysis, life expectancy included not just longevity but also quality of life and risk for premature death. Potential programs had to satisfy several criteria: (1) the factor was a likely driver of disparities in life expectancy based on previously published research, (2) the potential program was compatible with the strategic vision of WellSpan, (3) short-term outcomes could be quantified and measured, and (4) the program had the potential to have a strong impact on life expectancy. Factors considered for inclusion could involve WellSpan-provided health services (i.e., internal quality), or they could be community factors. Key factors identified by this initial analysis are listed in Table 3 according to their potential impact on life expectancy, quality of life, or premature death. The estimated impact was based on previously published studies. Future analyses may expand on this list.

**Table 3:** Factors affecting life expectancy, including longevity (L), quality of life (QoL), or risk of premature death (PD) in WellSpan’s service area.

Low impact	Medium impact	High impact
(QoL) Participation in Women, Infants, and Children (WIC)* (L) Routine annual well visit for people 65 years and older (L) Self-sufficiency programs (PD) Completion of recommended childhood vaccinations (PD) Participation in Systemic, Therapeutic, Assessment, Resources, and Treatment (START) (PD) Deaths attributed to violent crime*	(PD) Receiving medication-assisted treatment for substance use disorders, including opioid use disorder (L) Lung cancer screening PD) Lead testing in children (PD) Familial hyperlipidemia genetic testing (L) Publicly funded high quality pre-kindergarten* (PD) Housing Insecurity	(L) Colorectal cancer screening (L) Blood pressure control for people with uncontrolled hypertension (L) Breast cancer screening (L) Glycemic control

There may be overlap in types of impact; for example, factors affecting PD may also affect QoL. * Identified by analysis of community-based, publicly reported data. QoL, affects quality of life; L, affects longevity; PD, affects risk of premature death.

These factors and their estimated impacts were used to prioritize candidate programs for inclusion in WellSpan’s 30-year strategic plan to improve quality of life and reduce disparities in life expectancy and quality of life within our community.

## 6 Lessons learned and recommendations for other health systems

As WellSpan’s program evolved, we learned that development and implementation of CHIPs arising from the CHNA were top priorities, especially the importance of the CHIP as an active tool rather than simply a regulatory requirement. We also learned the value of consistent program evaluation and subsequent adjustment. Furthermore, effective actions required structured processes, clear governance, and support from the executive team and board of directors. Clearly defining the health system’s role in community partnerships is vital. For example, defining its role as funder, convener, advocate, etc., allows the health system to plan large and complex interventions with limited resources, avoids duplicative efforts, and ensures alignment with community partners and the health system’s mission. Targeted interventions require an understanding of the non-medical factors that drive disparities in health outcomes and life expectancy. Such factors include language barriers, low education levels, limited access to care, and other social drivers of health. Addressing these factors requires engagement with the community in humble and culturally sensitive ways. Finally, having a long-term mission is necessary for sustained change.

Our recommendations for other health systems seeking to develop a similar program mirror many of these lessons. We recommend establishing a core strategic priority similar to WellSpan’s: to provide exceptional care for everyone, in all geographic regions (urban, suburban, rural), and inclusive of all demographics served by the health system. We also recommend to strive for going beyond compliance in the development of a CHIP based on an in-depth CHNA, and utilize diverse data sources to establish a dependable, robust, measurement system. Finally, build a strong and tested governance structure that includes lean methods for communication and problem solving, and adopt a model of health system roles and responsibilities for collaborating with community partners.

## 7 Conclusion

This paper has documented WellSpan Health’s multi-year journey developing and improving community health improvement plans (CHIPs) that emphasize the goal of reducing disparities in health outcomes in the communities served. The experience acquired and lessons learned have led to the development of a 30-year plan for improving life expectancy and quality of life, and for reducing disparities in these outcomes within our community. In subsequent papers, we plan to describe plans for program evaluation and share initial results of intermediate outcomes.

## Data Availability

The raw data supporting the conclusions of this article will be made available by the authors, without undue reservation.
