# Supplementary material for: Development of strategies to improve care for all in the communities served by a mid-size nonprofit health system in the United States
Source: Front Public Health. 2025 Aug 1;13:1614507. doi: 10.3389/fpubh.2025.1614507 (PMC12354654; doi:10.3389/fpubh.2025.1614507)
Supplement: Supplementary file 1 [file Data_Sheet_1.pdf]

Cichetti JR, et al.

Supplementary Material

Supplementary Figure 1. Age of death according to county of residence and self-reported racial and ethnic group in 5 counties served by WellSpan Health System.

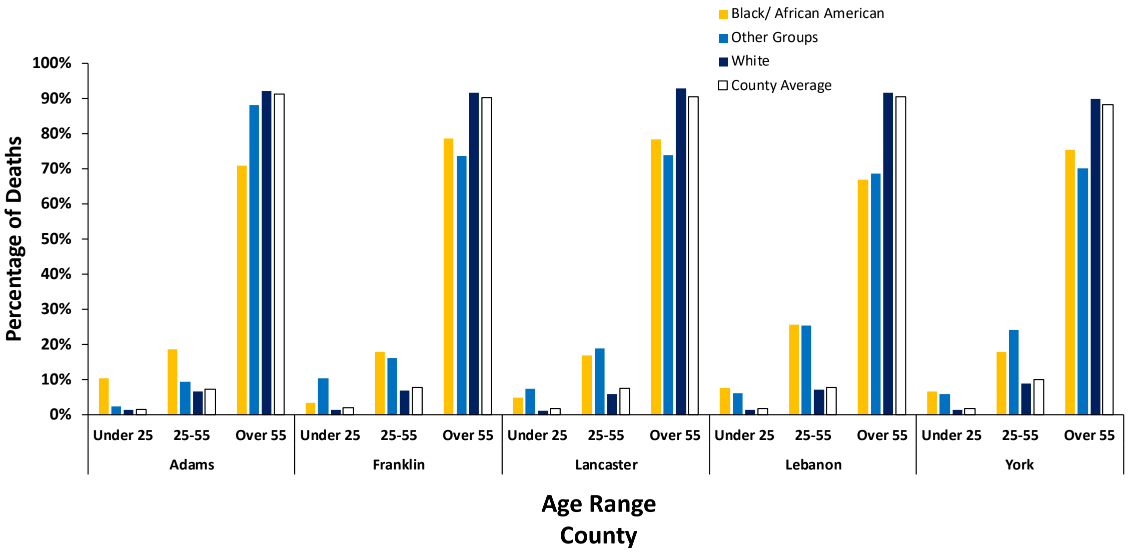

Supplementary Table 1. Maternal admissions and rates of severe maternal morbidity (SMM) at 4 WellSpan hospitals (York, Gettysburg, Ephrata and Good Samaritan). Statistical analysis by logistic regression.

Group	Year ending July 2019		July 2019-June 2022	
	Number of admissions	No. (%) with an SMM	Number of admissions	No. (%) with an SMM
**Total**	5124	37 (0.72)	22302	135 (0.61)
**Self-reported race**				
American Indian	11	0 (0)		
Asian	62	0 (0)	261	1 (0.4)
Black	343	7 (2.04)[a]	1314	17 (1.29)[b]
Pacific Islander	9	1 (11)		
White	3844	23 (0.60)	16129	79 (0.49)
Undetermined	96	3 (3)	310	2 (0.65)
**Self-reported Hispanic/Latino ethnicity**				
Yes	792	8 (1.01)	3642	32 (0.88)[c]
No	4233	28 (0.67)		

[a]P=0.00435 versus White patients
[b]P<0.001 versus White patients
[c]P=0.0051 versus non-Hispanic White patients

Supplementary Table 2. Rates of breast cancer screening mammography among eligible women before and after implementation of WellSpan's programs designed to increase screening rates.

	Total eligible (women 50-74 years of age)	Number (%) with screening mammography in preceding 27 months	Absolute change from baseline (%)
**Baseline (August 2021)**			
All eligible women	57648	40501 (70.26)	-
Women who self-identified as White non-Hispanic	52643	37084 (70.44)	-
Women who self-identified as American Indian, Asian, Black, non-White Hispanic, Pacific Islander, and other*	5005	3417 (68.27)	-
**June 2024**			
All eligible women	70760	53184 (75.16)	+4.90%
Women who self-identified as White non-Hispanic	63553	48010 (75.54)	+5.10%
Women who self-identified as American Indian, Asian, Black, non-White Hispanic, Pacific Islander, and other	7207	5174 (71.79)	+3.52%

*Some results are from older data sets in which racial and ethnic groups were combined. In the future, WellSpan intends to collect and record information for more narrowly defined groups.
